# How Does Aphasia Affect Quality of Life? Preliminary Reports

**DOI:** 10.3390/jcm12247687

**Published:** 2023-12-14

**Authors:** Karolina Filipska-Blejder, Jolanta Zielińska, Marek Zieliński, Adam Wiśniewski, Robert Ślusarz

**Affiliations:** 1 Neurological and Neurosurgical Nursing Department, Faculty of Health Science, Collegium Medicum in Bydgoszcz, Nicolaus Copernicus University in Toruń, Łukasiewicza 1 Street, 85-821 Bydgoszcz, Poland; 2 Faculty of Health Science, Cuiavian University in Włocławek, Plac Wolności 1 Street, 87-800 Włocławek, Poland; ziel.jolanta@gmail.com (J.Z.); mz1904@interia.pl (M.Z.); 3 Department of Neurology, Faculty of Medicine, Collegium Medicum in Bydgoszcz, Nicolaus Copernicus University in Toruń, Skłodowskiej 9 Street, 85-094 Bydgoszcz, Poland; adam.lek@wp.pl

**Keywords:** aphasia, ischemic stroke, quality of life

## Abstract

Aphasia leads to disability, which, in turn, limits and can result in a complete breakdown of roles and social bonds. Thus, the aim of this study was to assess the impact of aphasia disorders on the quality of life of patients after an ischemic stroke. A prospective study among 116 patients with an ischemic stroke (the study group: 68 participants, 38.25% female, included patients with aphasia after a stroke; the control group: 48 patients, 37.5% female, without aphasia) was conducted at the Neurological Department of the Provincial Specialist Hospital in Włocławek. The patients were assessed twice: the first assessment was conducted during hospitalization using the Aphasia Dynamics Assessment Scale and the Aphasia Test Method of Jadwiga Szumska, and the second assessment of the quality of life after six months was conducted using the WHOQOL-BREF questionnaire. The patients from the control group rated their overall quality of life more highly than the patients from the study group. Statistically significant differences were observed in the physical domain, the psychological domain, and the environmental domain. The analysis of sociodemographic factors indicated a negative impact on the quality of life of the following variables: female gender, 55–64 years of age, vocational education, and place of residence—rural area. The degree and type of aphasia influenced the overall quality of life. Studies with larger samples are necessary.

## 1. Introduction

According to the American Heart Association/American Stroke Association, “stroke is classically characterized as a neurological deficit attributed to an acute focal injury of the central nervous system (CNS) by a vascular cause, including cerebral infarction, intracerebral hemorrhage (ICH), and subarachnoid hemorrhage (SAH), and is a major cause of disability and death worldwide” [1]. There are two types of strokes: ischemic stroke (when a clot obstructs the flow of blood to the brain) and hemorrhagic stroke (when a blood vessel ruptures, preventing blood flow to the brain). Strokes area leading cause of long-term self-care deficits and morbidity. Moreover, it is the second most common cause of death in the world [2].

Language ability allows us to understand and express written and spoken words. The area of the brain responsible for language is usually located in the dominant hemisphere. These structures include Broca’s area, Wernicke’s area, and the arcuate fasciculus. Furthermore, speech distinguishes humans from other mammals. It is not only the key to communication and knowledge but is also an important factor that shapes personality [3,4,5]. Strokes are the most common cause of speech disorders like dysarthria and language disorders like aphasia. While both dysarthria and aphasia can be consequences of a stroke, they are distinct impairments and can co-occur [6]. Articulation disorders dominate in dysarthria, caused by muscle problems. Nevertheless, dysarthria does not in itself affect a person’s understanding. Aphasia is an impairment of the formulation or comprehension of language, which occurs in 21% to 38% of patients in the acute period of a stroke [7,8,9]. There are three main types of aphasia: global aphasia (the most severe form, extremely limiting the ability to comprehend language or speak); Broca’s aphasia (characterized by a significant limitation of speech, where statements are short and the patient often cannot find the right words); and Wernicke’s aphasia (impairment of the ability to grasp the meaning of spoken words) [3,4].

In light of the World Health Organization’s findings, it should be assumed that aphasia is not only a disorder of the ability to use language and speech but is also a disorder of the ability to communicate [10]. Moreover, the aphasia syndrome is integrally related to reading disorders (alexia), writing disorders (agraphia), and numeracy disorders (acalculia) [11,12]. Unfortunately, the above factors make aphasia an extremely severe disability among stroke patients, leading to a complete breakdown of social participation and deteriorating the quality of life [13]. Furthermore, speech disorders limiting mental and communicative efficiency should be treated on an equal footing with motor deficits, which significantly limit the motor efficiency of stroke patients [13,14,15,16,17]. Both of these stroke complications require immediate action and interdisciplinary management to minimize the risk of disability and the dependence of patients. Sensorimotor dysfunction prevents and complicates even simple movements and functional activities. Motor dysfunction mainly affects the upper and lower limbs that are contralesional to the damaged hemisphere. Moreover, during a stroke, oral motor disorders, muscle weakness, abnormalities in muscle tone, synchronization, and coordination are observed, in which a speech deficit is secondary to neurological damage [18,19,20].

Research conducted by Rangamani and Judovsky [21] confirmed the significant impact of cognitive deficits, aphasia severity, and therapy received on the quality of life. In turn, Bullier et al. [22] showed that fatigue, the severity of aphasia, functional dysfunctions, and mood disorders may negatively affect the quality of life of patients with aphasia. Additionally, Preetha and Perumal [23] assessed the impact of speech and language intervention on the quality of communication for persons with expressive aphasia. Patients undergoing therapy obtained higher scores in general domains compared to patients without speech therapy interventions. In fact, further research will be needed to help us understand and evaluate the impact of aphasia on patients’ quality of life across all domains. For this reason, the main aim of this study was to assess the impact of aphasia disorders on the quality of life of patients after an ischemic stroke. This study set the following specific objectives:

- How does aphasia affect the quality of life in persons after an ischemic stroke?
- Do selected sociodemographic factors influence the quality of life of the respondents?
- Does the type and degree of aphasic disorders affect the quality of life of stroke patients?

## 2. Materials and Methods

### 2.1. Participants and Settings

**Figure 1:**
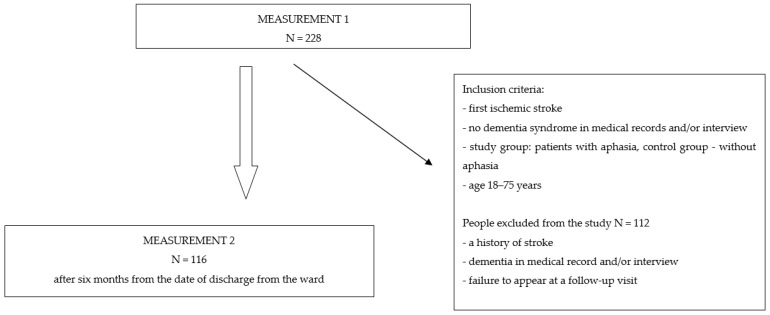
Selection of the study participants.

Prospective studies involving a twofold assessment of patients with an ischemic stroke using standardized tools were carried out at the Neurological Department of the Provincial Specialist Hospital of the Blessed Priest Jerzy Popiełuszko in Włocławek. This study included people who met the inclusion criteria: age 18–75 years, first ischemic stroke, and no dementia syndrome in medical records and/or interview. The study group comprised patients with aphasia and the control group patients without aphasia, with voluntary consent to participate in this study. Of the 228 patients who initially qualified for this study, a total of 116 remained, including 68 patients with aphasia in the study group and 48 patients in the control group without aphasia, six months after discharge from the ward. The reason for excluding 112 patients was their failure to appear at a follow-up visit. The selection of patients is presented in the diagram below (Figure 1). Both in the control group and in the study group, the largest percentage were men, aged 65–75, people with a vocational education, married, and living in the city. In all, 45.6% of the patients in the study group were diagnosed with mixed aphasia, followed by Broca’s (33.8%), global (11.8%), and Wernicke’s (8.8%). Moreover, 33.8% were individuals with moderate aphasia, 30.9% had mild aphasia, 23.5% had severe aphasia, and 11.8% had global aphasia. The overall data of the included patients are shown in Table 1.

The clinical diagnosis of an ischemic stroke was made by a neurologist. According to the criteria adopted in Poland, brain and neurovascular imaging is required for diagnosis. The current standard is a non-contrast computed tomography (CT) of the head and magnetic resonance imaging (MRI). Patients included in the study were admitted with acute symptoms of a stroke. The standard procedure in Poland is to perform a CT scan on the day of admission to the ward and then perform an MRI during the 9-day hospitalization in the ward. The diagnosis of aphasia was made by a speech pathologist. The first measurement performed during hospitalization consisted of a neurological diagnosis using standardized tests such as the Aphasia Dynamics Assessment Scale—SODA—and the Aphasia Test Method of Jadwiga Szumska. The second measurement, performed 6 months after discharge from the ward, consisted of assessing the quality of life using the WHOQOL-BREF questionnaire. The second assessment was performed during the follow-up visit. Patients completed the WHOQOL-BREF scale independently and could ask the researcher questions at any time.

### 2.2. Variables and Measurements

The following diagnostic tools were used for neurologopedic assessment:

– The Aphasia Dynamics Assessment Scale (SODA) [24]—a diagnostic questionnaire for aphasia that determines both the type of aphasia and its severity. Performing the test correctly requires at least two attempts. The average test time is approximately 5 min. The method of examining language disorders in the first days of a stroke should be short, simple, and not burdensome to the patient. The survey using the SODA scale meets the above conditions, and the short duration of the survey does not cause fatigue among respondents. The patient is assessed in three categories:
  A. Understanding simple verbal messages (e.g., close your eyes or open your mouth), 2-step verbal commands (close your eyes and open your mouth) and commands requiring understanding the structure of inflected sentences;
  B. The expression of speech demonstrated by the ability to provide personal data, automated word sequences, a repetition of simple words and sentences, dialogues, and descriptive speech;
  C. Naming objects known to the patient when shown by the speech pathologist.

Letter markings A, B, and C indicate the type of aphasia disorders, with A below 3 points indicating sensory aphasia, B below 3 points indicating motor aphasia, and C below 3 points indicating amnestic aphasia. In turn, the following criteria were adopted to assess the degree of disorders: 0–0.5 points, complete aphasia; 1–3.5 points, severe aphasia; 4–6.5 points, moderate aphasia; 7–8.5 points, mild aphasia; and 9 points, no features of aphasia [25].

– The Aphasia Test Method of Jadwiga Szumska [26]—considered an auxiliary tool intended for the diagnosis and assessment of language disorders in people with aphasia. This method is described in two parts: the first part is a theoretical introduction, while the content in the second part includes drawings, letters, numbers, etc., and is research material. It allows the clinician to indicate and distinguish the symptoms of speech disorders and to connect them to the brain damage described in the first part. This method uses the examination of descriptive speech, dialogues, and automated word sequences; a repetition study; a naming study; a speech understanding test; a reading examination; a writing examination; a counting examination; an examination of spatial orientation; and testing of the memory and praxis.

The following tools were used to assess the quality of life:

– The WHOQOL-BREF questionnaire of Krystyna Jaracz [27]—this 26-item scale is used to assess the quality of life in the following areas: physical (seven items; questions 3, 4, 10, and 15–18), psychological (six items; questions 5–7, 11, 19, and 26), social relationships (three items; questions 20–22), and environment functioning (eight items; questions 8, 9, 12–14, and 23–25). The following are subject to individual patient assessment:
  - In the physical domain (domain 1—DOM1): activities of daily living, the ability to work, energy and fatigue, mobility, dependence on medications and treatments, pain and discomfort, and rest and sleep;
  - In the psychological domain (domain 2—DOM2): appearance, negative feelings, positive feelings, self-esteem, spirituality, religion, personal faith, thinking, learning, memory, and concentration;
  - In the domain of social relationships (domain 3—DOM3): personal relationships, social support, and sexual activity;
  - In the functioning environment (domain 4—DOM4): financial resources, physical and mental safety, freedom, health and health care (availability and quality), opportunities to acquire new information and skills, home environment, opportunities and participation in recreation and relaxation, physical environment (pollution, noise, climate, and traffic), and transport.

In addition, WHOQOL-BREF also assesses the following:

- The quality of one’s life (question 1—WHO1);
- Individual overall perception of one’s health (question 2—WHO2).

The answers are measured on a 5-point scale (score range 1–5). The results of individual areas are positive (the higher the number of points, the higher the quality of life). In the research conducted by Jaracz et al. [27], the internal consistency for the total scale was 0.90. Furthermore, the internal consistency of the physical, psychological, social relationships, and environmental domains, assessed by Cronbach’s alpha, were 0.81, 0.78, 0.69, and 0.77, respectively.

Two questionnaires were also used in this study, one for the study group and the other for the control group. They contain questions about sociodemographic data such as gender, age, education, marital status, place of residence, and professional activity. The data sheet for the study group additionally contains questions allowing us to collect information about the speech therapy care facility as well as the duration of therapy and its frequency. The respondent also answered questions about linguistic communication: whether they experience difficulties in pronouncing words, problems with the verbalization of thoughts, difficulties in fully understanding verbal messages, and difficulties in the sphere of nominative speech.

### 2.3. Ethical Statement

This study was approved by the Bioethics Committee of the Nicolaus Copernicus University in Torun at Collegium Medicum of Ludwik Rydygier in Bydgoszcz, Poland (KB No. 774/2015, approval date 17 December 2015). This study was conducted according to the Declaration of Helsinki regarding research on humans. All subjects provided informed consent to participate in this study. In addition, they were informed about the purpose of this study and the possibility of resigning from participation at any stage.

### 2.4. Statistical Analysis

The statistical analysis was performed with STATISTICA version 13.1 (Dell Technologies, Round Rock, TX, USA). Hypotheses about the normality of distributions were verified using the Shapiro–Wilk and David Hellwig tests (compliance test). The significance of differences in mean values in two groups was verified with the chi-square test for unequally large groups. The chi-square test served to check the statistical equivalence of the groups (test group to control group) and to determine the significance level of the verified hypothesis. The significance of differences in mean values was checked using an analysis of variance. In the case of statistically significant ANOVA results, post hoc tests were performed (multiple comparisons using Fisher’s Least Significant Difference—LSD). Differences between variables were verified using non-parametric tests. Two independent groups were compared using the Mann–Whitney U test, and the Kruskal–Wallis H test was used to compare more than two independent groups. Statistical results with *p* < 0.05 were considered significant.

## 3. Results

The data analysis indicated that patients from the control group rate their overall quality of life higher than patients from the aphasia group (mean 3.27 vs. 2.85, *p* = 0.008, respectively). An assessment of the quality of life in individual areas was also carried out. Based on the data obtained, we concluded that the highest quality of life in both the aphasia group (mean 12.75) and the control group (mean 13.19) was in the field of social relations and the lowest in the field of environment (test group, mean 11.65; control group, mean 11.86). A comparison of the results of the questionnaire domains indicated that there is a statistically significant difference between the aphasia and control groups in the three domains examined, physical (*p* = 0.04), psychological (*p* = 0.02), and environmental (*p* = 0.04), and in the assessment of the overall quality of life (*p* = 0.008). In the social domain, only statistically significant differences between the compared groups were noted on the question regarding sexual activity (*p* = 0.017), which confirms the negative impact of aphasia on this area of the quality of life (Table 2).

Table 3 presents an assessment of the impact of sociodemographic factors on the quality of life, a self-assessment of health, and functioning in the physical, mental, social, and environmental fields, depending on variables such as gender, age, education, marital status, place of residence, and professional activity. The analysis of the collected material allowed us to conclude that gender has an impact on the general perception of the quality of life of the respondents only among women in the study groups. A statistically significant difference was observed only in the assessment of the overall quality of life (*p* = 0.013) and the self-assessment of health (*p* = 0.003) between the women of the study and control groups. This means that women after a stroke with aphasia rate their quality of life (mean 2.73) as lower than women after a stroke without aphasia (mean 3.28), and their health condition is rated as slightly higher (mean 2.31) than women from the control group (mean 2.22). Moreover, the analysis of the data clearly shows that statistically significant differences in the assessment of the quality of life of the study groups depending on age were observed only in the 55–64-year age group. These differences were noted in the overall quality of life (*p* = 0.047), in the field of physical fitness (*p* = 0.029), and in the psychological field (*p* = 0.001). This means that in this age range, stroke patients with aphasia evaluate their quality of life, as well as their physical and psychological functioning, as lower than stroke patients without aphasia. In the remaining cases, no statistically significant differences were noted. The impact of education on the quality of life of the respondents was also assessed. Based on the results obtained, we concluded that vocational education statistically significantly differentiates the quality of life of patients in the study and control groups only in the psychological field (mean 11.69 vs. 13.11, *p* = 0.020, respectively). Moreover, the analysis of the data clearly shows that the same statistically significant differences were noted in the overall quality of life among married patients and unmarried patients (single) (*p* = 0.020). This means that, in terms of the overall quality of life, married and unmarried patients after a stroke without aphasia indicate a higher quality of life. Significant differences were also noted among married patients of both groups (*p* = 0.003) in the psychological field, which also indicates a higher quality of life among patients from the control group. Studies have shown that people after an ischemic stroke with aphasia living in rural areas have a lower quality of life in the psychological field than people from the control group (mean 12.00 vs. 13.80, *p* = 0.024, respectively). Moreover, the obtained results clearly show that professional activity statistically significantly differentiates the quality of life of respondents only in the psychological field and only in working respondents (*p* = 0.021).

The quality of life was also assessed for individual types of aphasia in the study group. As Table 3 shows, significant differences were found in patients with total aphasia (*p* = 0.048), motor aphasia (*p* = 0.045), and sensory aphasia (*p* = 0.032) for the overall quality of life. In the psychological field, a statistically significant difference was observed only in patients with motor and sensory aphasia. This type of aphasia affects the overall quality of life as well as the psychological domain. The quality of life was also assessed for individual degrees of aphasia in the study group. A statistically significant difference was observed in the overall quality of life in patients with complete, severe, and moderate aphasia. In the self-assessment of health, a statistically significant difference was found only in patients with total aphasia. In the physical, psychological, and environmental domains, a statistically significant difference was observed in patients with severe aphasia. The deeper the degree of aphasia, the lower the overall quality of life and self-assessment of health and physical, psychological, and environmental functioning. No statistically significant difference was observed among patients only in the area of social relationships (Table 3).

## 4. Discussion

In recent years, increasing attention has been focused on assessing the quality of life. Along with the effectiveness of therapy, the improvement in the quality of the patient’s functioning and their subjective assessment of their situation are important elements. Attention to quality is a necessary requirement in the diagnostic and therapeutic process because negligence in this area threatens the highest values: human health and life [28]. So far, no universal, quick, short, standard measurement tool to assess the quality of life has been developed, as indicated by Kłak et al. [29], who stated that examining the level of the quality of life in medical sciences is a holistic approach to the patient. The multiplicity of tools may, therefore, cause difficulties in comparing results and generalizing conclusions. Despite the complexity of the subject and methodology in terms of the quality of life, we attempted to assess the impact of the type and degree of aphasic disorders on the quality of life of patients after an ischemic stroke. To our knowledge, this is one of the few studies in Poland assessing in such detail the quality of life related to individual aphasic disorders. Conducting research in this area is also important because nearly half of stroke patients struggle with post-stroke aphasia (PSA). Moreover, research indicates that aphasia significantly affects all language functions, well-being, and the quality of life [30]. In our project, a lower quality of life was observed among patients with aphasia six months after the onset of a stroke. This means that stroke patients with aphasia perceive their functioning to be lower than patients from the control group. Additionally, many stroke patients have neurological deficits that cause difficulties in concentrating, thinking, learning, and, above all, memory, which may significantly affect their assessment of the quality of life, but these aspects were not assessed in our project. In the study conducted by Jarosławski et al. [31], the quality of life was assessed using the European Quality of Life Scale–5 Dimensions EQ-5D-3L (EQ-5D) among 172 patients more than six months after the occurrence of an ischemic stroke. The leading factors influencing all dimensions of the EQ-5D scale include depression and speech problems. Moreover, research shows that mainly depressive disorders and aphasia worsen the quality of life after a stroke. Interestingly, there are studies that link the risk of depression with PSA, such as that of Lin et al. [32], who assessed the risk of depression in patients with aphasia after a stroke. The median of the observation period was 7.91 years and 8.62 years in the group of subjects with aphasia (N = 26,754) and without aphasia (N = 139,102). It was observed that depressive disorders occurred more often in people with aphasia than without aphasia (9.02 vs. 8.13 per 1000 person-years). Additionally, people with PSA, regardless of gender and the type of stroke, have been shown to experience depression more often. Moreover, in the meta-analysis by Cai et al. [33], a significant positive relationship was found between depressive disorders and the risk of a stroke (hazard ratios: 1.39; 95% CI: 1.22–1.58; *p* < 0.001). Thus, it is important to conduct PSA tests and to introduce modern treatment therapies.

Our analysis of the quality-of-life assessment in individual areas indicates that the highest quality-of-life assessment in both the study group (mean 12.75) and the control group (mean 13.19) was recorded in the area of social relations, and the lowest was recorded in the area of the environment (study group, mean 11.65; control group, mean 11.86). We noted identical results in the research of Weber-Rajek M. et al. [34], where, on the one hand, social support, including family and friends, may have a mitigating effect on the consequences of a stroke and can lead to an improvement in the quality of life, and, on the other hand, it may cause post-stroke depression. Welten et al. [35] examined the strength of the relationship between a stroke patient and their partner. They found that the patient’s proactive coping and functioning correlate positively with a lower level of anxiety in the partner. In addition, a greater sense of self-efficacy in the partner translates into lower rates of depression and provides a greater sense of satisfaction for the patient. Comparing the differences in our own research between the average values for individual questions in the field of social relations in the study and control groups, we noted that significant differences occurred only in sexual activity (*p* = 0.017). Weber-Rajek et al. [34] also noted in their research that, despite the highest values obtained in the field of social relations, only single people evaluate their quality of life in social relations as much lower, especially in sexual activity. Furthermore, Kauhanen [36] noted that sexual disorders, including a decreased libido and sexual arousal, as well as a dissatisfaction with their sexual life, seem to be common in patients after a stroke. King [37] also reported a decrease in sexual satisfaction in stroke patients in his studies covering many years.

The results of our study confirm the impact of the type of aphasia on the overall quality of life as well as on the psychological domain. Additionally, a statistically significant difference was noted in the overall quality of life in patients with complete, severe, and moderate aphasia. Bachia and Chun [38] found that people with non-fluent aphasia rate their quality of life as poorer than people with fluent aphasia. The three most affected domains are language, social roles, and thinking. Vuković [39] also noted that patients with a milder aphasia rated their quality of life as higher in most domains. Spaccavento et al. [40] observed that patients with acute aphasia assessed communications, autonomy, and the total quality of life on the questionnaire for aphasia as poorer than patients with chronic aphasia.

A limitation of this study is the lack of reports from similarly designed studies, which would allow for a more detailed analysis of our results. It is only possible to compare our results with those of authors conducting research using other tools. Another significant limitation is the too-small group of people examined, which does not allow for drawing clear conclusions regarding the quality of life of people after an ischemic stroke with aphasia in Poland. Additionally, complete data on patients in the control group was not obtained (the problems that its participants have after a stroke). However, our study indicates the need to conduct this type of research on a much larger scale, allowing us to assess the role and impact of speech therapy on the quality of life in patients after an ischemic stroke with aphasia.

## 5. Conclusions

Patients after an ischemic stroke with aphasia evaluate their overall quality of life and their physical, psychological, and environmental functioning as lower than that of patients after an ischemic stroke without aphasia. However, they do not show differences in their self-assessment of health and social relationships. Moreover, it was shown that the type and degree of aphasic disorders affect the quality of life of patients. Each type of aphasia worsens the overall quality of life, and, additionally, motor and sensory aphasias negatively affect psychological functioning. We also found that the more advanced the degree of aphasic disorders, the lower the overall quality of life and self-assessment of health and functioning in the physical, psychological, and environmental fields.

## Figures and Tables

**Table 1:** Sociodemographic characteristics of the participants.

Characteristics	Aphasia (N = 68)	Control (N = 48)	Total (N = 116)	Statistical Analysis
	N (%)	N (%)	N (%)	p
Sex				
Female	26 (38.2)	18 (37.5)	44 (37.9)	0.07
Male	42 (61.8)	30 (62.5)	72 (62.1)	
Age				
<54 years	4 (7.4)	3 (6.3)	7 (6.1)	0.91
55–64 years	23 (33.8)	18 (37.5)	41 (35.3)	
65–75 years	41 (60.3)	27 (56.2)	68 (58.6)	
Education				
Primary	5 (7.4)	3 (6.3)	8 (6.9)	0.78
Secondary	20 (29.4)	16 (33.3)	36 (31.0)	
Vocational	37 (54.4)	27 (56.2)	64 (55.2)	
Higher	6 (8.8)	2 (4.2)	8 (6.9)	
Marital status				
Widow/Widower	14 (20.6)	8 (16.7)	22 (19.0)	0.70
Married	46 (67.6)	32 (66.6)	78 (67.2)	
Single, Divorcee	8 (11.8)	8 (16.7)	16 (13.8)	
Place of residence				
Village	23 (33.8)	17 (35.4)	40 (34.5)	0.86
City	45 (66.2)	31 (64.6)	76 (65.5)	
Professional activity				
Unemployed	4 (5.9)	5 (10.4)	9 (7.7)	0.67
Pensioner	46 (67.6)	31 (64.6)	77 (66.4)	
Worker	18 (26.5)	12 (25.0)	30 (25.9)	
Type of aphasia				
Global	8 (11.8)	-	-	-
Mix	31 (45.6)	-	-	
Broca’s (non-fluent)	23 (33.8)	-	-	
Wernicke’s (fluent)	6 (8.8)	-	-	
The degree of aphasia				
Global	8 (11.8)	-	-	-
Severe	16 (23.5)	-	-	
Moderate	23 (33.8)	-	-	
Mild	2 (30.9)	-	-	

**Table 2:** Quality of life assessed by WHOQOL-BREF questionnaire.

WHOQOL-BREF	Aphasia (N = 68)		Control (N = 48)		Statistical Analysis	
	Mean	SD	Mean	SD	Z	p
Q1 Overall quality of life	2.85	0.80	3.27	0.87	−2.64	0.008 *
Q2 Self-assessment of health condition	2.39	0.73	2.46	0.71	−0.80	0.42
DOMAIN 1 Physical Health	11.66	3.15	12.46	2.85	−1.79	0.04 *
DOMAIN 2 Psychological	11.70	3.15	12.76	2.85	−2.32	0.02 *
DOMAIN 3 Social relationships	12.75	3.40	13.19	3.40	−0.74	0.46
DOMAIN 4 Environment	11.65	2.57	11.86	2.13	−2.02	0.04 *

Note. *—significant dependencies, p—Mann–Whitney U test, SD—Standard Deviation.

**Table 3:** Characteristics of individual WHOQOL-BREF domains in the study and control groups based on demographic variables and the type and degree of aphasia.

Characteristic	A (N = 68)	C (N = 48)		A (N = 68)	C (N = 48)		A (N = 68)	C (N = 48)		A (N = 68)	C (N = 48)		A (N = 68)	C (N = 48)		A (N = 68)	C (N = 48)	
	Q1 Overall Quality of Life			Q2 Self-Assessment of Condition			D1 Physical Health			D2 Psychological			D3 Social Relationships			D4 Environment		
	Mean (SD)	Mean (SD)	p	Mean (SD)	Mean (SD)	p	Mean (SD)	Mean (SD)	p	Mean (SD)	Mean (SD)	p	Mean (SD)	Mean (SD)	p	Mean (SD)	Mean (SD)	p
Sex ^1^																		
Female	2.73 (0.72)	3.28 (0.57)	0.01 *	2.31 (0.68)	2.22 (0.65)	0.003 *	11.45 (2.83)	11.62 (3.03)	>0.05	11.13 (1,76)	12.26 (2.60)	>0.05	12.67 (2.56)	12.96 (3.16)	>0.05	11.62 (2.57)	11.64 (1.89)	>0.05
Male	2.93 (0.84)	3.27 (1.01)	>0.05	2.43 (0.77)	2.60 (0.72)	>0.05	11.80 (3.36)	12.97 (2.65)	>0.05	12.05 (2.29)	13.07 (2.83)	>0.05	12.79 (3.86)	13.33 (3.59)	>0.05	11.68 (2.60)	12.00 (2.27)	>0.05
Age ^1^																		
<55	2.75 (0.50)	3.67 (0.58)	-	2.50 (0.58)	2.67 (0.58)	-	12.57 (1.92)	14.48 (1.19)	-	13.33 (2.11)	13.56 (2.69)	-	13.0 (4.41)	13.33 (4.0)	-	12.0 (4.14)	10.83 (2.75)	-
55–64	2.91 (0.90)	3.44 (0.86)	0.04 *	2.17 (0.83)	2.83 (0.51)	0.12	11.80 (3.76)	14.22 (2.15)	0.03 *	11.36 (2.37)	14.11 (2.29)	0.001 *	12.41 (3.89)	14.07 (3.55)	0.16	11.41 (2.85)	12.75 (1.59)	0.22
65–75	2.83 (0.77)	3.11 (0.89)	0.20	2.49 (0.68)	2.19 (0.74)	0.17	11.50 (2.90)	11.07 (2.62)	0.32	11.72 (1.97)	11.78 (2.70)	0.88	12.91 (3.09)	12.59 (3.25)	0.80	11.76 (2.30)	11.39 (2.24)	0.50
Education ^1^																		
Primary	2.60 (0.55)	3.00 (0.00)	-	1.80 (0.45)	1.67 (0.58)	-	10.40 (3.37)	8.00 (2.97)	-	10.93 (2.03)	10.67 (1.33)	-	12.27 (2.89)	13.33 (1.33)	-	11.00 (2.24)	10.50 (1.32)	-
Secondary	2.95 (0.82)	3.38 (1.09)	0.20	2.45 (0.96)	2.31 (0.70)	0.68	11.54 (3.25)	11.86 (2.76)	0.98	11.83 (2.97)	12.54 (3.32)	0.27	13.53 (4.77)	14.50 (2.92)	0.47	11.83 (3.67)	12.00 (2.22)	0.99
Vocational	2.86 (0.75)	3.22 (0.80)	0.70	2.41 (0.64)	2.31 (0.69)	0.16	11.92 (3.23)	13.44 (2.40)	0.55	11.69 (2.20)	13.11 (2.46)	0.02 *	12.22 (3.42)	12.30 (3.65)	0.83	11.46 (2.47)	11.96 (2.11)	0.50
Higher	2.67 (0.75)	3.50 (0.71)	-	2.50 (0.84)	2.50 (0.71)	-	11.52 (3.46)	10.86 (1.62)	-	11.89 (2.87)	13.00 (3.30)	-	13.78 (3.83)	14.67 (3.77)	-	12.83 (2.23)	11.50 (3.54)	-
Marital status ^1^																		
Single	2.50 (0.53)	3.25 (0.46)	0.20 *	2.00 (0.53)	2.50 (0.53)	0.08	12.07 (3.32)	13.21 (2.15)	0.22	10.83 (2.46)	12.33 (2.36)	0.15	8.17 (3.45)	10.00 (1.89)	0.07	10.25 (3.20)	11.00 (1.56)	0.40
Married	3.02 (0.83)	3.41 (0.95)	0.20 *	2.50 (0.81)	2.53 (0.72)	0.44	11.75 (3.27)	12.93 (2.83)	0.06	12.17 (1.96)	13.54 (2.62)	0.003 *	14.20 (2.54)	15.04 (2.01)	0.09	12.12 (2.41)	12.56 (1.94)	0.27
Widow/Widower	2.50 (0.65)	2.75 (0.71)	0.23	2.29 (0.47)	2.13 (0.83)	0.39	11.18 (2.69)	9.86 (2.20)	0.05	10.62 (1.95)	10.08 (1.87)	0.38	10.57 (1.92)	9.00 (3.00)	0.17	10.96 (2.35)	9.94 (1.97)	0.15
Place of residence ^1^																		
Village	2.96 (0.55)	3.41 (0.94)	0.70	2.39 (0.75)	2.59 (0.80)	0.36	11.70 (0.96)	13.24 (3.29)	0.11	12.00 (3.23)	13.80 (2.66)	0.02 *	13.45 (2.03)	14.20 (2.90)	0.40	12.17 (2.87)	12.71 (2.03)	0.37
Small city	2.83 (0.75)	3.25 (0.50)	-	2.67 (0.45)	2.50 (0.58)	-	13.90 (0.84)	13.71 (2.24)	-	13.11 (3.25)	13.67 (1.76)	-	15.11 (2.20)	15.00 (2.96)	-	14.00 (2.89)	12.13 (2.17)	-
Big city	2.79 (0.82)	3.19 (0.88)	0.08	2.33 (0.64)	2.37 (0.69)	0.80	11.30 (3.37)	11.79 (2.51)	0.74	11.30 (3.46)	11.98 (2.73)	0.24	11.97 (2.97)	12.30 (3.57)	0.69	10.99 (3.42)	11.30 (2.07)	0.82
Professional activity ^1^																		
Unemployed	2.50 (0.58)	3.40 (0.55)	-	2.00 (0.82)	2.80 (0.45)	-	11.00 (3.04)	13.71 (1.14)	-	10.50 (1.99)	13.20 (2.42)	-	6.33 (2.75)	10.40 (2.19)	-	9.50 (2.74)	10.00 (0.94)	-
Pensioner	2.80 (0.86)	3.16 (0.86)	0.80	2.41 (0.72)	2.23 (0.72)	0.37	11.19 (3.10)	11.37 (3.52)	0.98	11.59 (1.96)	12.00 (3.84)	0.40	12.93 (2.88)	12.73 (4.75)	1.00	11.54 (2.45)	11.55 (3.63)	1.00
Worker	3.06 (0.64)	3.50 (1.00)	0.09	2.39 (0.78)	2.92 (0.51)	0.07	13.02 (3.05)	14.76 (2.07)	0.11	12.22 (2.54)	14.56 (2.12)	0.02 *	13.70 (3.42)	15.56 (2.37)	0.17	12.42 (2.66)	13.46 (1.16)	0.82
Type of aphasia ^2^																		
Global	2.63 (0.74)	-	0.04 *	2.00 (0.53)	-	0.08	11.36 (4.30)	-	0.37	11.42 (3.22)	-	0.24	13.00 (3.00)	-	0.78	11.31 (2.69)	-	0.69
Mix	3.03 (0.75)	-	0.14	2.65 (0.66)	-	0.48	11.96 (2.60)	-	0.51	12.19 (1.77)	-	0.24	12.77 (3.39)	-	0.61	11.95 (2.10)	-	0.84
Broca’s (non-fluent)	2.78 (0.90)	-	0.04 *	2.17 (0.83)	-	0.23	11.68 (3.48)	-	0.61	11.45 (2.08)	-	0.03 *	12.58 (3.96)	-	0.56	11.63 (2.95)	-	0.83
Wernicke’s (fluent)	2.50 (0.55)	-	0.03 *	2.33 (0.52)	-	0.56	10.48 (3.29)	-	0.11	10.44 (2.22)	-	0.04 *	12.89 (2.18)	-	0.62	10.67 (3.46)	-	0.35
The degree of aphasia ^2^																		
Global	2.63 (0.74)	-	0.01 *	2.00 (0.53)	-	0.04 *	11.36 (4.30)	-	0.37	1.42 (3.22)	-	0.24	13.00 (3.00)	-	0.78	11.31 (2.69)	-	0.69
Severe	2.69 (0.60)	-	0.01 *	2.31 (0.60)	-	0.41	10.61 (2.29)	-	0.03 *	11.33 (2.18)	-	0.04 *	11.67 (4.20)	-	0.20	10.50 (2.85)	-	0.004 *
Moderate	2.74 (0.92)	-	0.03 *	2.39 (0.84)	-	0.80	11.88 (3.33)	-	0.69	12.03 (2.03)	-	0.21	12.70 (3.24)	-	0.53	12.00 (2.72)	-	0.45
Mild	3.19 (0.75)	-	0.53	2.57 (0.75)	-	0.74	12.35 (3.02)	-	0.88	11.71 (1.81)	-	0.07	13.52 (3.05)	-	0.83	12.29 (1.93)	-	0.51

Note. *—significant dependencies, ^1^—Mann–Whitney U test, ^2^—Kruskal–Wallis test, A—Aphasia group, C—Control group.

## Data Availability

The data presented in this study are available on request from the corresponding author. The data are not publicly available due to respondents privacy.
